# Integrated transcriptomic and proteomic analyses of plerocercoid and adult *Spirometra mansoni* reveal potential important pathways in the development of the medical tapeworm

**DOI:** 10.1186/s13071-023-05941-8

**Published:** 2023-09-05

**Authors:** Rui Jie Wang, Wen Li, Shi Nan Liu, Si Yao Wang, Peng Jiang, Zhong Quan Wang, Xi Zhang

**Affiliations:** Department of Parasitology, School of Basic Medical Sciences, Zhengzhou University, Zhengzhou, 450001 Henan China

**Keywords:** *Spirometra mansoni*, Plerocercoid, Differentially expressed proteins, Comparative proteome, Integrated transcriptomic and proteomic analysis

## Abstract

**Background:**

*Spirometra mansoni* can parasitize animals and humans through food and water, causing parasitic zoonosis. Knowledge of the developmental process of *S. mansoni* is crucial for effective treatment; thus, it is important to characterize differential and specific proteins and pathways associated with parasite development.

**Methods:**

In this study, we performed a comparative proteomic analysis of the plerocercoid and adult stages using a tandem mass tag-based quantitative proteomic approach. Additionally, integrated transcriptomic and proteomic analyses were conducted to obtain the full protein expression profiles of different life cycle stages of the tapeworm.

**Results:**

Approximately 1166 differentially expressed proteins (DEPs) were identified in adults versus plerocercoids, of which 641 DEPs were upregulated and 525 were downregulated. Gene Ontology (GO), Clusters of Orthologous groups (COG) and Kyoto Encyclopedia of Genes and Genomes (KEGG) analyses indicated that most DEPs related to genetic information processing and metabolism of energy in adults seem to be more activated. In the plerocercoid stage, compared to metabolism, genetic information processing appears more dynamic. Protein-protein interaction (PPI) revealed six key proteins (phosphomannomutase, glutathione transferase, malate dehydrogenase, cytoplasmic, 40S ribosomal protein S15, ribosomal protein L15 and 60S acidic ribosomal protein P2) that may play active roles in the growth and development of *S. mansoni*. Finally, the combination of transcriptomic and proteomic data suggested that three pathways (ubiquitin-mediated proteolysis, phagosome and spliceosome) and five proteins closely related to these pathways might have a significant influence in *S. mansoni*.

**Conclusions:**

These findings contribute to increasing the knowledge on the protein expression profiles of *S. mansoni* and provide new insights into functional studies on the molecular mechanisms of the neglected medical tapeworm.

**Graphical Abstract:**

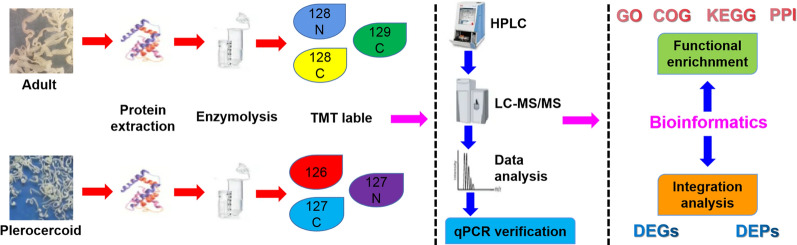

**Supplementary Information:**

The online version contains supplementary material available at 10.1186/s13071-023-05941-8.

## Background

Parasitic tapeworms can cause major socioeconomic impacts; however, many tapeworms have been substantially neglected in terms of research and control, such as *Spirometra mansoni* (Cestoda: Diphyllobothriidae) [1, 2]. The plerocercoid (sparganum) of *S. mansoni* can parasitize humans and animals, causing a food/water-borne parasitic zoonosis known as sparganosis [3]. The occurrence of human sparganosis has been reported in Asian, African, American and European countries, with more than 2000 cases to date [3–5]. Humans are usually infected by drinking water with infected copepods, consuming raw or uncooked meat infected with plerocercoids (e.g., tadpoles, frogs or snakes) and directly contacting raw meat of secondary hosts used as a poultice through eyes or wounds [6, 7]. Research on *S. mansoni* has long been neglected, and knowledge of the molecular biological basis for *S. mansoni* is fragmentary. Thus, few methods for the diagnosis and treatment of sparganosis have been developed. Therefore, addressing this knowledge gap is an urgent matter. Fortunately, the development of omics technology provides a good opportunity to further clarify the molecular mechanisms of organisms.

In recent years, with the continuous development of omics, some achievements have been made in research on *Spirometra* tapeworm at the molecular level. The first draft genome of *Spirometra erinaceieuropaei* using plerocercoid tissue was assembled in 2014 [8], and it was updated using the resequencing method to perform a comparative genomic analysis with the cryptic parasite *Sparganum proliferum* in 2021 [9]. Both studies provided valuable genomic information to obtain an in-depth understanding of the molecular characteristics of *Spirometra* tapeworms. In 2020, a phosphoproteomic analysis of *S. erinaceieuropaei* plerocercoids was performed to acquire knowledge on the protein phosphorylation networks of *Spirometra* tapeworms [10]. More recently, a comparative transcriptome analysis between adult and larval stages of *S. erinaceieuropaei* was conducted to better characterize differential and specific genes and pathways associated with parasite development [11]. The comparative transcriptional dataset provided valuable clues for the biological and physiological mechanisms behind the development and reproduction of this neglected zoonotic parasite. In contrast to transcriptomic data, proteomics is a systematic analysis of proteins that can provide a more complete description of global functional protein expression in cells, tissues and body fluids, providing a wealth of information not available by other methods [12]. Additionally, proteomics can characterize both proteomes and sub-proteomes (subcellular proteomics is a proteomics study aimed at the structural and functional units of different regions in the cell) without knowledge of the nature of proteins and can discover new targets, which is a major advantage [13, 14]. It also provides an opportunity to study protein expression, protein interactions and protein modifications [15]. For cestode parasites, several pioneering works have been completed using proteomic data. For example, a comparative proteomic analysis of two consecutive developmental stages of *Hymenolepis diminuta* (cysticercoid and adult) was performed to distinguish proteins that might be characteristic of each stage [16]. In 2018, a proteomic approach was used to describe and compare the larval tetrathyridium and adult protein repertories of *Mesocestoides corti* for the development of novel diagnostic methods and therapeutic drugs for cestodiases [17]. For *Echinococcus* tapeworms, the hydatid fluid (HF) composition of two *E. multilocularis* isolates (EmH95 and EmG8065) was compared through liquid chromatography coupled with tandem mass spectrometry (LC-MS) to explore potential protein targets for the diagnosis and treatment of alveolar echinococcosis [18]. The proteomes of two developmental stages of *Echinococcus granulosus* were analysed by using the isobaric tag for relative and absolute quantitation (iTRAQ) approach in 2013 and updated using the two-dimensional LC-MS method in 2021. Both studies provided new insights into the molecular mechanisms of host-parasite interactions [19, 20]. In addition, proteomic analysis of the hydatid fluid of *E. granulosus* and *Echinococcus ortleppi* revealed important mechanisms related to basic cellular processes and functions that act at the host-parasite interface in cystic echinococcosis [21]. Within *Taenia* tapeworms, the quantitative multiplexed proteomics of *T. solium* cysts indicated the occurrence of tissue-enriched antigen, which could be useful in the improvement of the immunodiagnosis for cysticercosis [22]. The protein profiling of *Taenia ovis* metacestodes determined by LC-MS/MS provided clues for better understanding the molecular basis of *T. ovis* [23]. However, in contrast to those of taeniid tapeworms, no proteomic data are available regarding the different lifecycle stages of *Spirometra* tapeworms. Therefore, we intended to explore the proteomic profiles of *S. mansoni* at different developmental stages as well as to further investigate its molecular characteristics using the integrated dataset of proteomes and transcriptomes.

In this study, we performed a comparative proteomic analysis between adults and plerocercoids of *S. mansoni* based on tandem mass tags (TMTs). TMT is a multiplex technique that enables high-efficiency and high-throughput relative quantification of proteins in multiple biological samples in a single mass spectra run and is widely used in proteomics studies [24]. More specifically, the following objectives were addressed: (i) to identify and characterize differentially expressed proteins (DEPs) between the plerocercoid and adult stages of *S. mansoni* and (ii) to integrate transcriptomic and proteomic analyses to fully reveal the protein expression profiles of different lifecycle stages of the tapeworm to provide more valuable clues for understanding the molecular biological basis of *S. mansoni*.

## Methods and materials

### Ethics statement

This study was performed strictly based on the recommendations of the Guide for the Care and Use of Laboratory Animals of the National Health Commission of China. The protocol was approved by the Life Science Ethics Committee of Zhengzhou University (permission no. 2020-0704).

### Samples and experimental animals

**Fig. 1:**
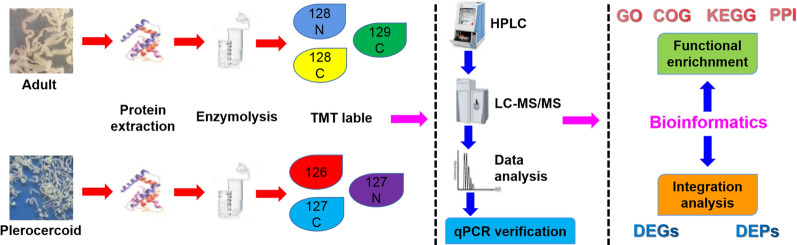
Flow chart of the whole study. Adult and plerocercoids were prepared. Protein extraction, enzymatic hydrolysis and TMT labelling were carried out. Peptide fragments were analyzed by LC-MS/MS. Q-PCR quantitative verification and bioinformatics analysis of differentially expressed proteins were performed

The plerocercoids were collected from infected frogs (*Pelophylax nigromaculatus*) from Zhengzhou city in central China [25] and identified as *S. mansoni* by molecular typing using the method described in Kuchta et al. [3]. The head of the plerocercoid was used to infect cats and obtain adult worms, and the body part was used for proteomic sequencing. As described previously, an adult cestode was obtained from an infected domestic cat [7]. All samples were washed thoroughly with physiological saline, snap-frozen in liquid nitrogen and stored at − 80 °C for further use. The whole process of our experiment is shown in Fig. 1.

### Protein extraction and quantification

Protein was extracted from pooled plerocercoids (*n* = 3) or pooled adults (gravid segment, *n* = 3) using the following process. First, each sample was ground into powder and dissolved in the appropriate UA extraction buffer (Amresco). Then, the mixture was sonicated on ice for 2 min at 1-s pulse intervals; debris was removed by centrifugation at 14,000 × g for 20 min, and the resulting supernatant was collected. Ten microlitres of supernatant was used for quantification. The concentration of extracted protein was determined by using the Bradford method (Pierce™ Thomas Plus assay kit, Thermo Fisher Scientific) according to the manufacturer's instructions.

### Trypsin digestion and TMT labelling

Reduction of the sulfhydryl bonds was performed using 5 mM DTT in 25 mM ammonium bicarbonate (Sigma–Aldrich) at 37 °C for 1 h and alkylation of sulfhydryl with 10 mM iodoacetamide (Amresco) at room temperature for 45 min in the dark. The solution was enzymatically digested using trypsin (Promega, Germany) at a ratio of 50:1 (protein/enzyme) at 37 °C overnight. The samples were desalted using a C18 column and dried down. Within the TMT labelling experiment, the samples were labelled with the TMT10plex™ Isobaric Label Reagent Set (Thermo Fisher Scientific) in triplicate (plerocercoid: 126, 127N, 127C; adult worm: 128N, 128C, 129C). The TMT reagent was first concussed for 5 min, centrifuged with 41 μl of acetonitrile and then mixed with 100 μg of digested samples at room temperature for 1 h. The reaction was terminated by adding ammonia water (Wako Pure Chemical Industries Ltd.).

### High-performance liquid chromatography fractionation and LC-MS/MS

The mixed labelled samples were fractionated by RIGOL L-3000 high-performance liquid chromatography (HPLC, Beijing Puyuan Precision Technology) using an Acclaim PepMap100 column (4 cm × 100 μm, C18, 150/100 μm) (Thermo Fisher Scientific) and a two-mobile-phase gradient elution system (mobile phase A: 100% water, 0.1% formic acid; mobile phase B: 80% acetonitrile, 0.1% formic acid). The fraction gradient was as follows: 0–5 min 5–8% B, 5–40 min 8–18% B, 40–62 min 18–32% B, 62–64 min 32–95% B, 64–68 min 95% B and 68–72 min 95–5% B. The flow rate was 0.7 ml/min. Subsequently, 1 µg samples dissolved in mobile phase A were centrifuged at 14,000 × g for 20 min at 4 °C and loaded onto a Q Exactive HF-X mass spectrometer (Thermo Fisher Scientific) with an XBridge Peptide BEH5 µm C18 column (Waters). The elution gradient was 0–7 min 7–15% B, 7–34 min 15–25% B, 34–49 min 25–40% B, 49–50 min 40–100% B and 50–60 min 100% B. The flow rate was 600 nl/min. Then, peptides were analysed by liquid chromatography coupled with tandem mass spectrometry (LC-MS/MS) using higher energy C-trap dissociation (HCD), positive ionization mode and a data-dependent acquisition (DDA) strategy, which involved full-spectrum MS mode and full-spectrum product-ion (MS-MS) analysis mode. The settings of the first-stage mass spectrum were as follows: NSI voltage, 2.4 kV; capillary temperature, 275 ℃; scan range, 407–1500 m/z; resolution, 60,000 at m/z 200; automatic gain control (AGC) target, 3 × 106; maximum injection time of C-trap, 20 ms. MS/MS acquisition targeted the 40 most intense parent ions. The settings for the secondary mass spectrum were as follows: resolution, 4500 at m/z 200; AGC target, 5 × 104; maximum injection time, 86 ms; peptide fragmentation collision energy, 32%. Then, the raw proteomic data were generated (Additional file 1).

### Database search

Raw MS data files were analysed using Proteome Discoverer 2.4 (Thermo Fisher Scientific). The retrieval parameter settings were as follows: the database was UniProt *Spirometra erinacei* UP000550142 (Additional file 3: Table S1); the cleavage enzyme digestion method was set as Trypsin/P with no more than two missing cleavages, and the minimum peptide length was 7; the tolerance of precursor ion mass was set to 15 ppm; the tolerance of mass error of secondary fragment ions was 0.02 Da. Carbamidomethyl (C) was set as a fixed modification, variable modification as M Oxidation (15.995 Da) and TMT-16plex (K, N-terminal) acetylation of protein. The protein quantification method was set with TMT 16-plex. The reporter ion isotopic impurity distribution of TMT 16-plex was set as the product manual. Protein identification and FDR by PSM identification were set as 1%. The mass spectrometry proteomics data were deposited at the ProteomeXchange Consortium (http://proteomecentral.proteomexchange.org) via the iProX partner repository [26, 27] with the dataset identifier PXD039620.

### Bioinformatics

The Gene Ontology (GO) annotation was derived from the UniProt-GOA database (http://www.ebi.ac.uk/GOA/). The proteins were classified by GO annotation (http://geneontology.org/) according to biological process (BP), cellular component (CC) and molecular function (MF) categories. The Kyoto Encyclopedia of Genes and Genomes (KEGG) database (https://www.genome.jp/kegg/) was used to annotate the protein pathway based on the Python and Fisher’s exact test. COG (Clusters of Orthologous Groups)/KOG (Clusters of euKaryotic Orthologous Groups) functional classification of the identified proteins was conducted through database comparison and analysis (https://www.ncbi.nlm.nih.gov/research/cog/). Differential analysis was performed using a *t* test. According to previous studies, proteins with a *P* < 0.05 and an absolute fold change (FC) ≥ 1.5 were considered differentially abundant [28]. Volcano plots and heatmaps of differentially expressed proteins (DEPs) were generated using the R language pheatmap and ggplot2 packages, respectively. The enrichment analysis of the DEPs against all identified proteins was tested using the Fisher’s exact test to calculate *p* values. Multiple hypothesis testing was further applied to obtain the Qvality q-value (FDR). The smaller the *p* value or FDR, the higher the enrichment degree. In addition, the description of significantly differentially expressed proteins from plerocercoids and adults was searched against the database of the STRING [29] (https://www.string-db.org/) protein network, and then the differentially screened protein-protein interaction (PPI) relationships were extracted according to a confidence score > 0.7 (high confidence). Furthermore, the degree of each protein was calculated to evaluate the importance of the protein in the PPI network. The interaction network from STRING was visualized in Cytoscape [30].

### Quantitative real-time PCR verification

The candidate proteins were verified by confirming the transcription levels identified using qRT-PCR. A total of 12 proteins (6 upregulated and 6 downregulated proteins) were randomly selected and measured. Total RNA of plerocercoids and adults was extracted using TRIzol reagent (Invitrogen, USA) based on the manufacturer’s instructions. RNA was dissolved in RNase-free ddH2O (Takara, China), and cDNA was synthesized using a reverse transcription kit (Novoprotein, Shanghai, China). The reverse transcribed first-strand cDNA was used as a template for real-time PCR. Primers used for RT-PCR were synthesized by Sangon Biotech (Shanghai, China) (Additional file 2: Table S2). RT-PCR was performed on an Applied Biosystems 7500 Fast Real Time PCR System (Applied Biosystems, USA). GAPDH was used as a housekeeping gene. Statistical differences between two groups were analysed using Student’s t-test with significant differences at *P* < 0.05.

### Correlation analysis of transcriptomic and proteomic data

To comprehensively understand the differences in molecular characteristics of genes related to growth and development between adults and plerocercoids, transcriptome-proteome correlation analysis was performed by selecting the most significantly different genes and proteins based on GO annotation and KEGG enrichment results. For the transcriptomic data, although transcriptomes of both adult and plerocercoid *Spirometra* tapeworms have been published [11], we still performed a resequencing analysis of transcriptomes using specimens in this study for the following reasons: (i) the transcriptomic data generated in Liu et al. [11] were based on a de novo assembly method because the previously published draft genome of *S. erinaceieuropaei* (Bioproject ID PRJEB1202) was incomplete [8]; however, an updated genome (Bioproject ID PRJEB35375) was released recently [9], so we conducted a map-based assembly using the new reference genome based on the similarity value (above 70%). (ii) We ensured the coherence and consistency of different omic data to avoid unnecessary systematic errors during the analysis. RNA-seq and proteomic analyses were conducted with three independent biological replicates. The Pearson correlation coefficient (PCC) of DEGs and DEPs was calculated to divide genes and proteins into the same or opposite expression trend using the cor function in the R package (*P* < 0.05 represented significance, *P* < 0.01 represented high significance, and *P* < 0.001 represented extreme significance). A heatmap was used to show the correlations between the *S. mansoni* transcriptome and proteome, and a pathway enrichment analysis was performed to screen out the pathways that may play important roles in *S. mansoni* based on the KEGG database.

## Results

### Quantitative proteomic analysis by TMT labelling

**Fig. 2:**
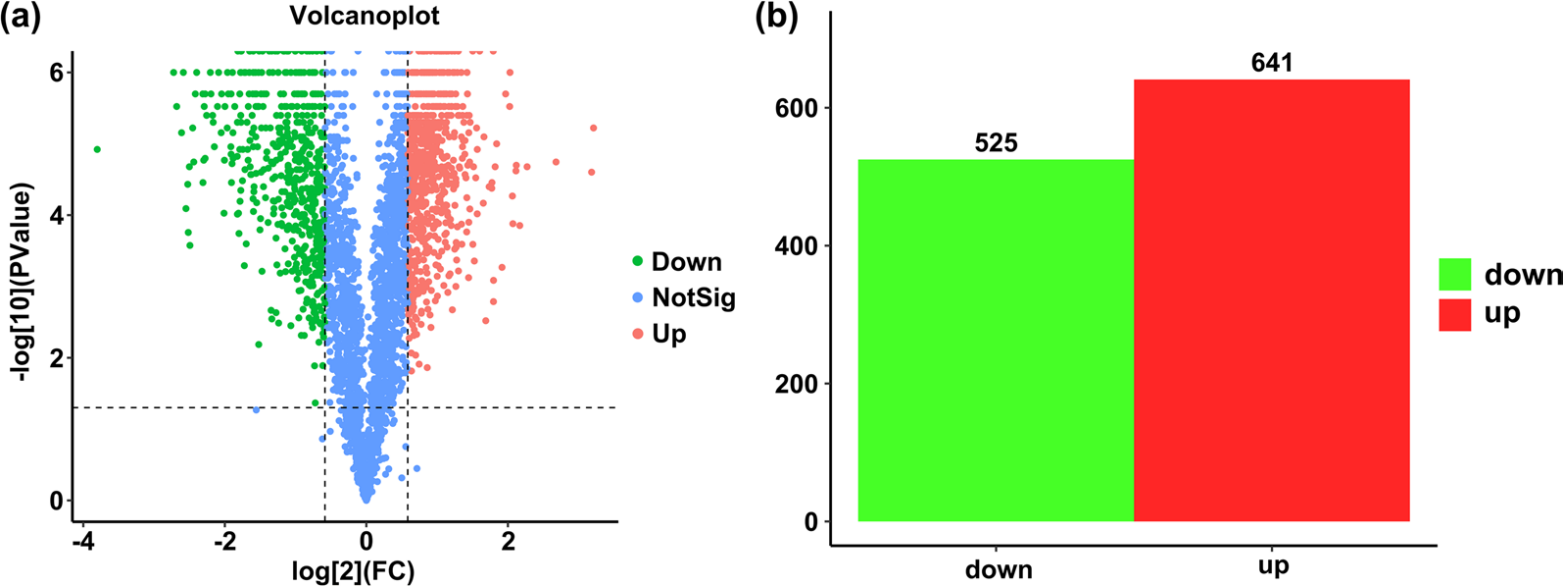
Differentially expressed protein (DEP) profiles by TMT-based proteomic analysis. **a** Volcano plot of differentially expressed proteins between adult and plerocercoid stages. Differential expression level of protein: red dot indicates significant upregulation of protein, green dot indicates significant downregulation of protein, and blue indicates that the protein expression level is not statistically significant. **b** Quantitative distribution of differentially expressed proteins between adult and plerocercoid stages. Red indicates significant upregulation, and green indicates significant downregulation

Changes in the proteome between plerocercoids and adults of *S. mansoni* were evaluated by TMT labelling and quantified by liquid chromatography-mass spectrometry analysis (Fig. 1). Among a total of 249,937 spectra, 57,798 could be matched to the database, resulting in a total of 30,722 peptides assembled into 3378 protein groups with high confidence (FDR < 0.01, Additional file 4: Table S3). The numbers of proteins with a single peptide, 2–5 peptides, 6–10 peptides and ≥ 11 peptides were 876, 1349, 494 and 432, respectively (Additional file 2: Fig. S1). Volcano plots were examined to identify the proteins that were possibly responsible for the difference between adults and plerocercoids with a statistically significant difference (*P* < 0.05, and > 1.5 or < 0.66-fold change) (Fig. 2a). In the comparison, 1166 proteins were defined as DEPs between adults and plerocercoids, among which 641 and 525 proteins were upregulated and downregulated, respectively (Fig. 2b). The differentially expressed proteins in two developmental stages of *S. mansoni* are listed in Additional file 5: Table S4.

### GO enrichment analysis of DEPs

**Fig. 3:**
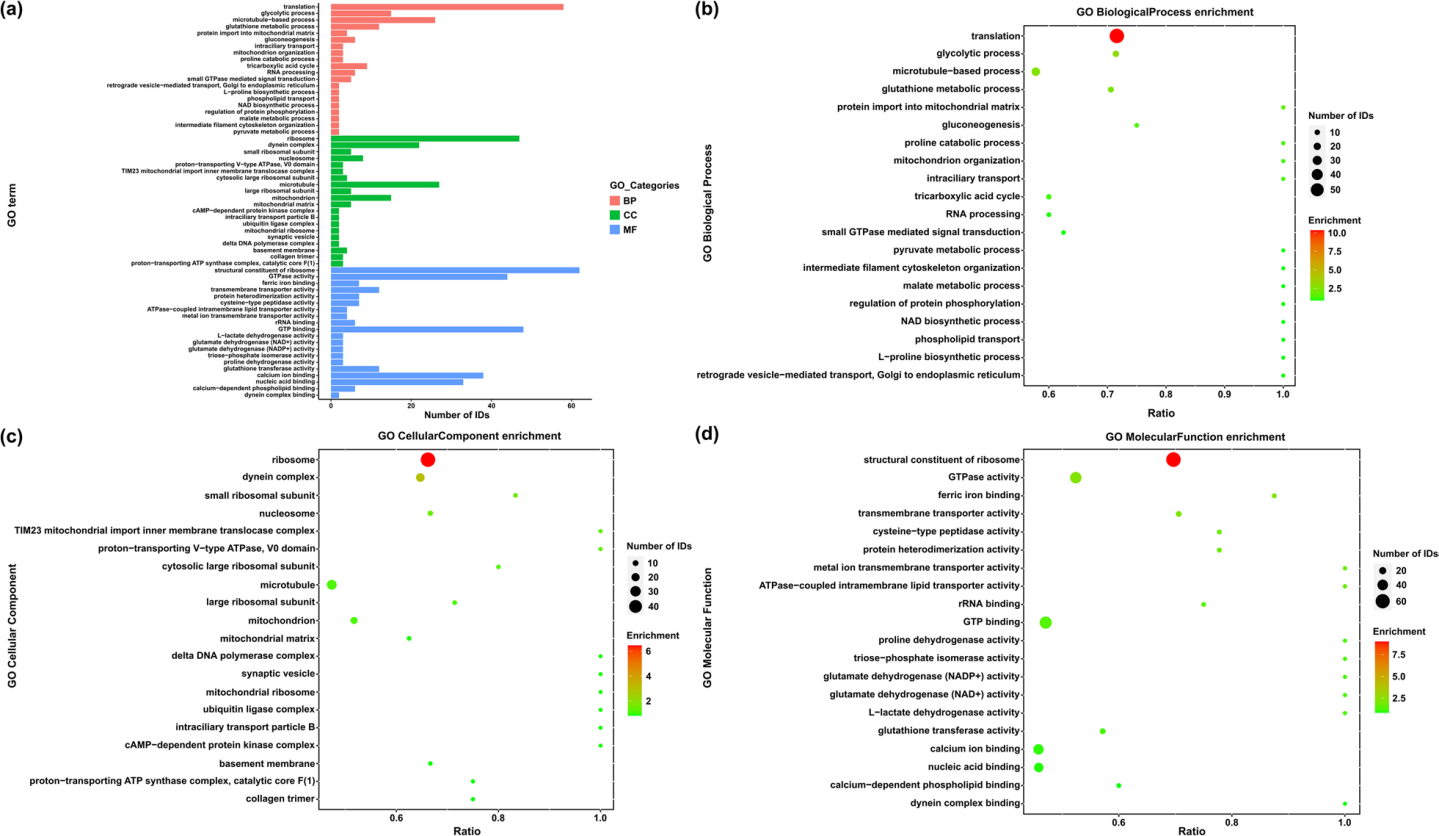
GO functional enrichment for the differentially expressed proteins. **a** The top 20 GO enrichments of significantly regulated proteins in adults. **b** Distribution of the top 20 most abundant Gene Ontology (GO) terms of significantly regulated proteins in the category of biological process. **c** Distribution of the top 20 most abundant Gene Ontology (GO) terms of significantly regulated proteins in the category of cellular component. **d** Distribution of the top 20 most abundant Gene Ontology (GO) terms of significantly regulated proteins in the category of molecular function

GO enrichment analysis was performed to identify processes enriched in the DEPs using the hypergeometric distribution test. Of the 1166 DEPs between adults and plerocercoids, 810 DEPs were successfully annotated with 692 GO terms. Among these DEPs, 451 DEPs were categorized into biological processes (BP), 491 were categorized into cellular components (CC), and 661 were categorized into molecular functions (MF) (Fig. 3a). In the BP category, most frequent GO terms were translation, microtubule-based process and glycolytic process (Fig. 3b). In the CC category, the following GO terms were abundant: ribosome, dynein complex and small ribosomal subunit (Fig. 3c). The most abundant terms in the MF category were structural constituent of ribosome followed by GTPase activity and ferric iron binding (Fig. 3d). Moreover, GO term analysis was carried out to evaluate significantly overrepresented GO terms to obtain a detailed view of stage-specific upregulated genes (Additional file 2: Fig. S2).

### COG analysis of DEPs

**Fig. 4:**
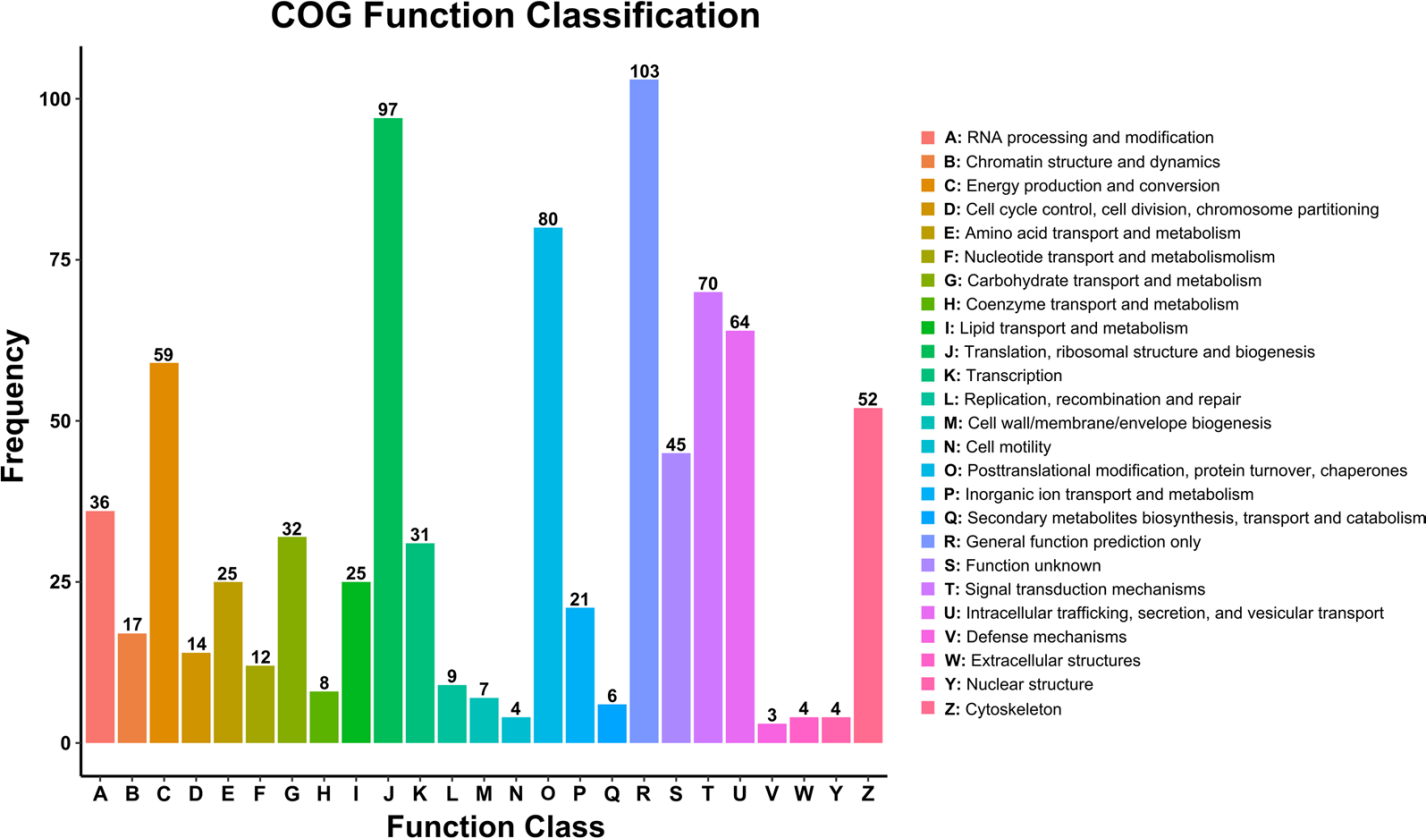
COG analysis of identified proteins in adults vs. plerocercoids. The X-axis represents classification, while the Y-axis represents the number of identified proteins

DEPs were also annotated using the COG database. As shown in Fig. 4, a total of 771 DEPs were classified into 25 categories. "General function prediction only" (103 DEPs, 47 downregulated and 56 upregulated) was the most abundant functional category followed by "translation, ribosomal structure and biogenesis" (97 DEPs, 2 downregulated and 95 upregulated), "posttranslation modification, protein turnover, chaperones" (80 DEPs, 36 downregulated and 44 upregulated) and "signal transduction mechanisms" (70 DEPs, 46 downregulated and upregulated). Based on these clusters, we identified the top 30 DEPs correlated with the production, transport and metabolism of nutrients in the growth and development of *S. mansoni* from the 771 DEPs, likely lipid transport and metabolism (phosphomannomutase), amino acid transport and metabolism (isocitrate dehydrogenase [NAD] subunit, mitochondrial), carbohydrate transport and metabolism [oxoglutarate dehydrogenase (succinyl-transferring)], nucleotide transport and metabolism (adenylosuccinate synthetase) and energy production and conversion (ADP-ribosyl cyclase/cyclic ADP-ribose hydrolase) (Additional file 2: Table S5).

### KEGG pathway enrichment analysis of DEPs

**Fig. 5:**
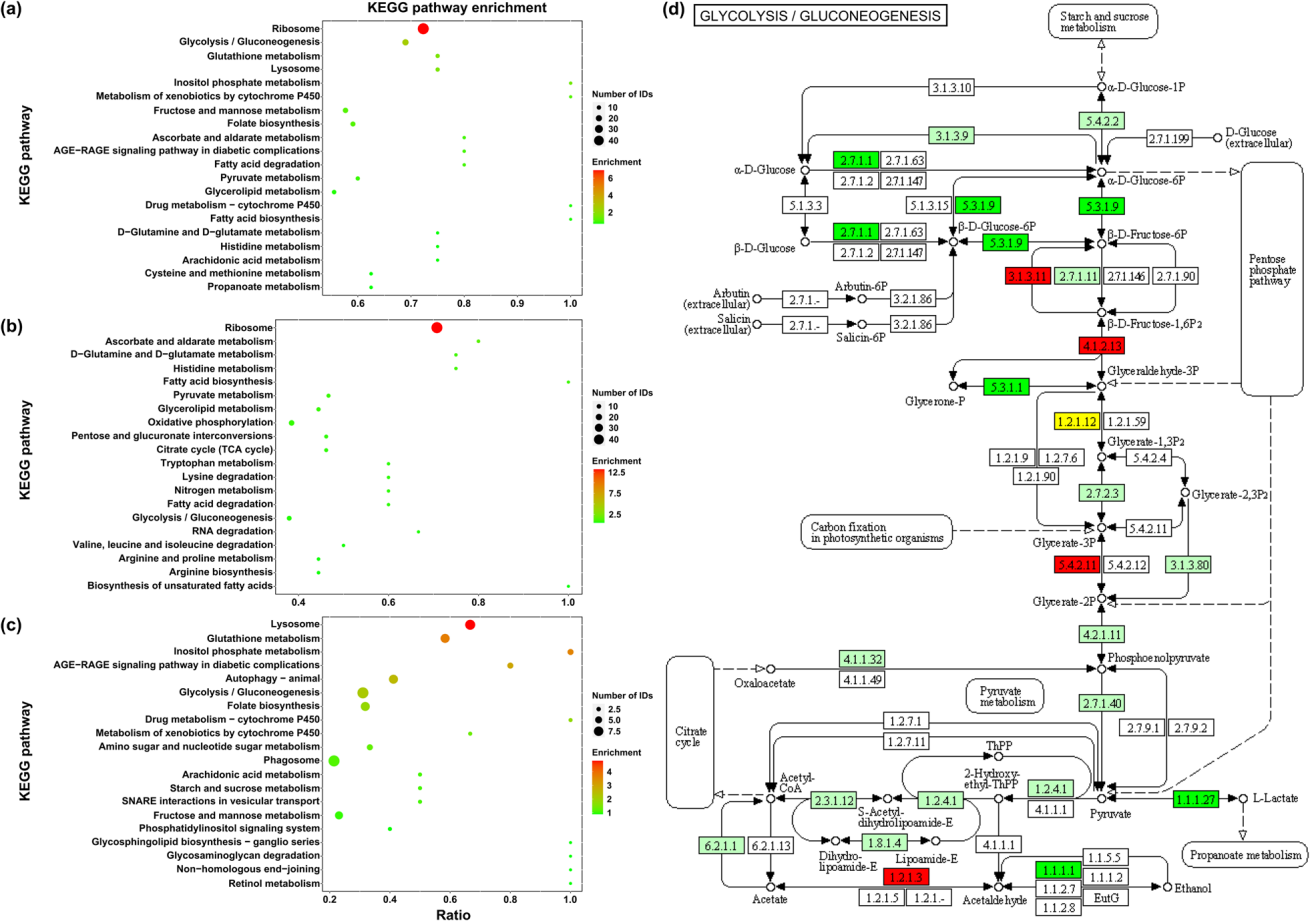
KEGG enrichment analysis of differentially expressed proteins in adults vs. plerocercoids. **a** Enrichment analysis of the 20 most significant KEGG pathways of differentially expressed *Spirometra mansoni* proteins between adults and plerocercoids. **b** Enrichment analysis of the 20 most significant KEGG pathways of upregulated proteins in adults. **c** Enrichment analysis of the 20 most significant KEGG pathways of upregulated proteins in the plerocercoid. **d** Representative KEGG pathway for glycolysis/gluconeogenesis. Twenty differentially expressed proteins of adults and plerocercoids were involved in the glycolysis/gluconeogenesis pathway. Red and green symbols represent proteins that were up- or downregulated in adults, respectively

To identify the changes in biological pathways operating during the two developmental stages, DEPs were mapped to reference pathways in the KEGG database. Of all 1166 DEPs between adults and plerocercoids, 190 differentially expressed proteins were enriched in 82 pathways. The most enriched KEGG pathway was ribosome, followed by glycolysis/gluconeogenesis, lysosome and glutathione metabolism (Fig. 5a). In addition, KEGG enrichment analysis was carried out to evaluate significantly overrepresented KEGG terms to obtain a detailed view of stage-specific upregulated proteins. A total of 132 upregulated proteins in adults were associated with 58 pathways. The highly enriched pathways included ribosome, ascorbate and aldarate metabolism and histidine metabolism (Fig. 5b). Regarding the plerocercoid stage, 58 upregulated proteins were associated with 54 pathways. The top three enriched enrichments were lysosome, glutathione metabolism and inositol phosphate metabolism (Fig. 5c). Moreover, a representative pathway map of glycolysis/gluconeogenesis is presented in Fig. 5d. Several important DEPs were founded in the pathway: fructose-bisphosphatase, fructose-bisphosphate aldolase, glyceraldehyde-3-phosphate dehydrogenase, triosephosphate isomerase, L-lactate dehydrogenase, phosphotransferase, glucose-6-phosphate isomerase and hypothetical protein. Among these DEPs, 9 proteins were upregulated in plerocercoids, and 11 proteins were upregulated in adults, indicating that the glycolysis/gluconeogenesis pathway is more active in adults and that adults probably need more energy during development and egg production.

### PPI analysis of DEPs

**Fig. 6:**
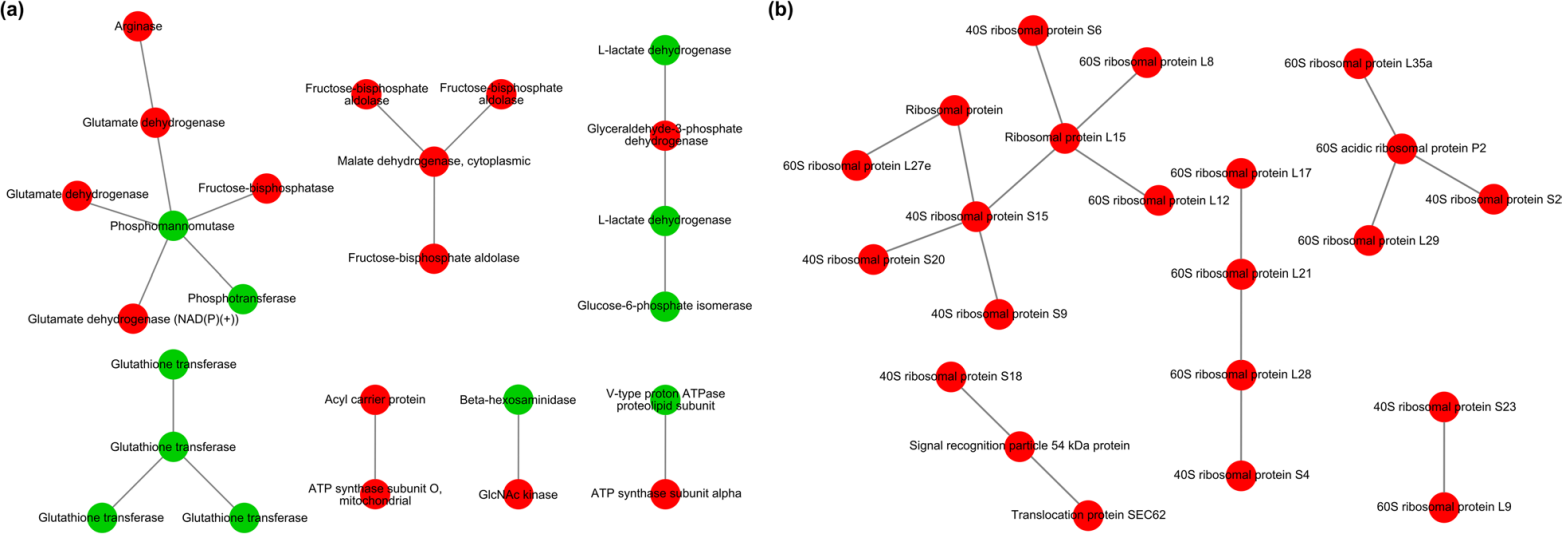
Protein–protein interaction network analysis. **a** PPI network of metabolic proteins. **b** PPI network of proteins related to genetic information processing. The circles in the figure represent differentially expressed proteins, with the downregulated proteins in green and upregulated proteins in red

A total of 89 proteins with descriptions and functional annotations were screened from all DEPs. Of these, 42 proteins were involved in metabolism, and 42 proteins were related to genetic information processing. To demonstrate the relationship of proteins in metabolic pathways and genetic information processing clearly, protein interaction network analysis was performed by submitting 84 proteins to the STRING database (Additional file 2: Tables S6 and S7). For proteins related to metabolism, the PPI network analysis showed that some DEPs interact with each other, such as arginase-glutamate dehydrogenase-phosphomannomutase-phosphotransferase and glucose-6-phosphate isomerase-L-lactate dehydrogenase-glyceraldehyde-3-phosphate dehydrogenase. In addition, the following important node proteins were identified: phosphomannomutase (5 nodes), glutathione transferase (3 nodes) and malate dehydrogenase, cytoplasmic (3 nodes) (Fig. 6a). These key focus hubs have important biological functions in fructose and mannose metabolism, arachidonic acid metabolism, glutathione metabolism, citrate cycle (TCA cycle) and pyruvate metabolism. For proteins related to genetic information processing, the PPI analysis showed that DEPs of 60S ribosomal protein L8-ribosomal protein L15-40S ribosomal protein S15-40S ribosomal protein S20 and 60S ribosomal protein L17-60S ribosomal protein L21-60S ribosomal protein L28-40S ribosomal protein S4 interacted with each other. There were three important node proteins: 40S ribosomal protein S15 (4 nodes), ribosomal protein L15 (4 nodes) and 60S acidic ribosomal protein P2 (3 nodes). These key focus hubs have important biological functions in ribosomes, structural constituents of ribosomes and translation (Fig. 6b). In summary, these important node proteins are mainly associated with the following categories: metabolism (especially in the metabolism of nutrients) and genetic information processing (especially in the regulation of translation). In plerocercoids, the major regulatory trend of metabolism was upregulated, and the major regulatory trend of genetic information processing was downregulated. In addition, PPI analysis suggested that changes in these high connectivity proteins might affect the growth and development of *S. mansoni*.

### Validation of the transcription of DEPs by qRT-PCR

**Fig. 7:**
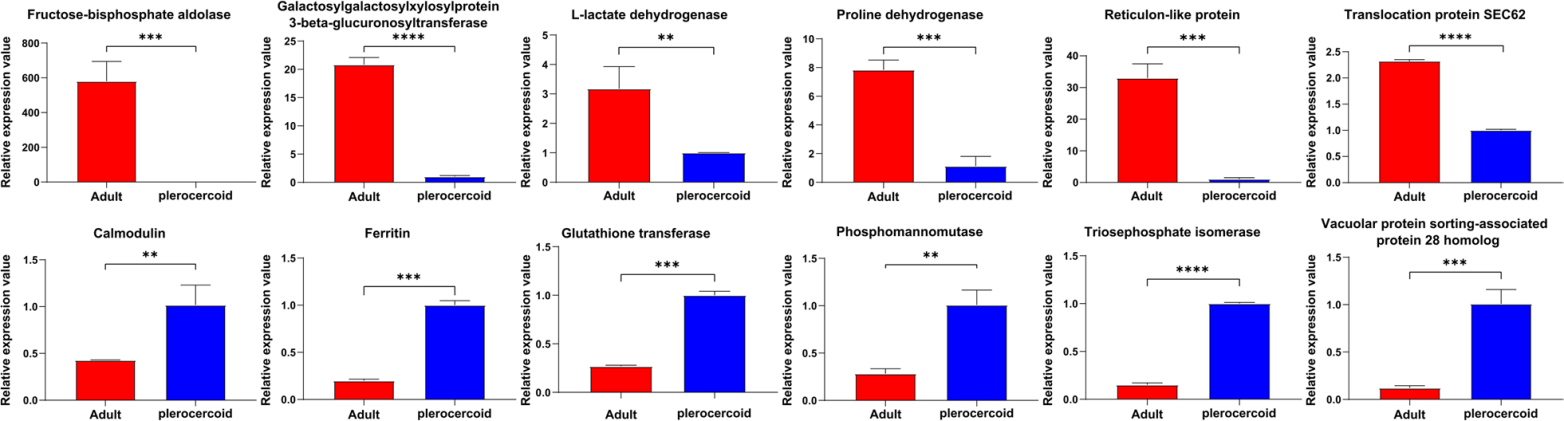
qPCR validation of differentially expressed *Spirometra mansoni* genes. **a** The mRNA expression levels were significantly higher in adults than in plerocercoids. **b** The mRNA expression levels were significantly higher in plerocercoids than in adults. GAPDH was used for normalization. The results are given as the mean ± SEM (standard mean of error) of samples (*n* = 3). Asterisks indicate significant differences (*P* < 0.05)

A total of 12 DEPs in each plerocercoid and adult were randomly selected for qRT-PCR analysis to validate the proteomic data (Fig. 7). The expression levels were calculated according to the 2^−ΔΔCt^ values. According to the sequencing results, the expression levels of A0A7M3R3A0 (L-lactate dehydrogenase), A0A7M3QRP6 (fructose-bisphosphate aldolase), A0A7M3REN6 (galactosylxylosylprotein 3-beta-glucuronosyltransferase), A0A7M3QU61 (translocation protein SEC62), A0A7M3RKT1 (reticulon-like protein) and A0A7M4CK85 (proline dehydrogenase) were upregulated in adults. The expression levels of A0A7M3Q4K1 (glutathione transferase), A0A7M3R222 (triosephosphate isomerase), A0A7M3QXI1 (vacuolar protein sorting-associated protein 28 homologue), A0A7M3RFA3 (phosphomannomutase), A0A7M3QKI6 (calmodulin) and A0A7M3RYR5 (ferritin) were upregulated in plerocercoids. Accordingly, the qRT-PCR analysis showed that the expression trends of the selected proteins were consistent with those obtained by proteomics, confirming the accuracy and reliability of the proteomic results.

### Correlation between DETs and DEPs

**Fig. 8:**
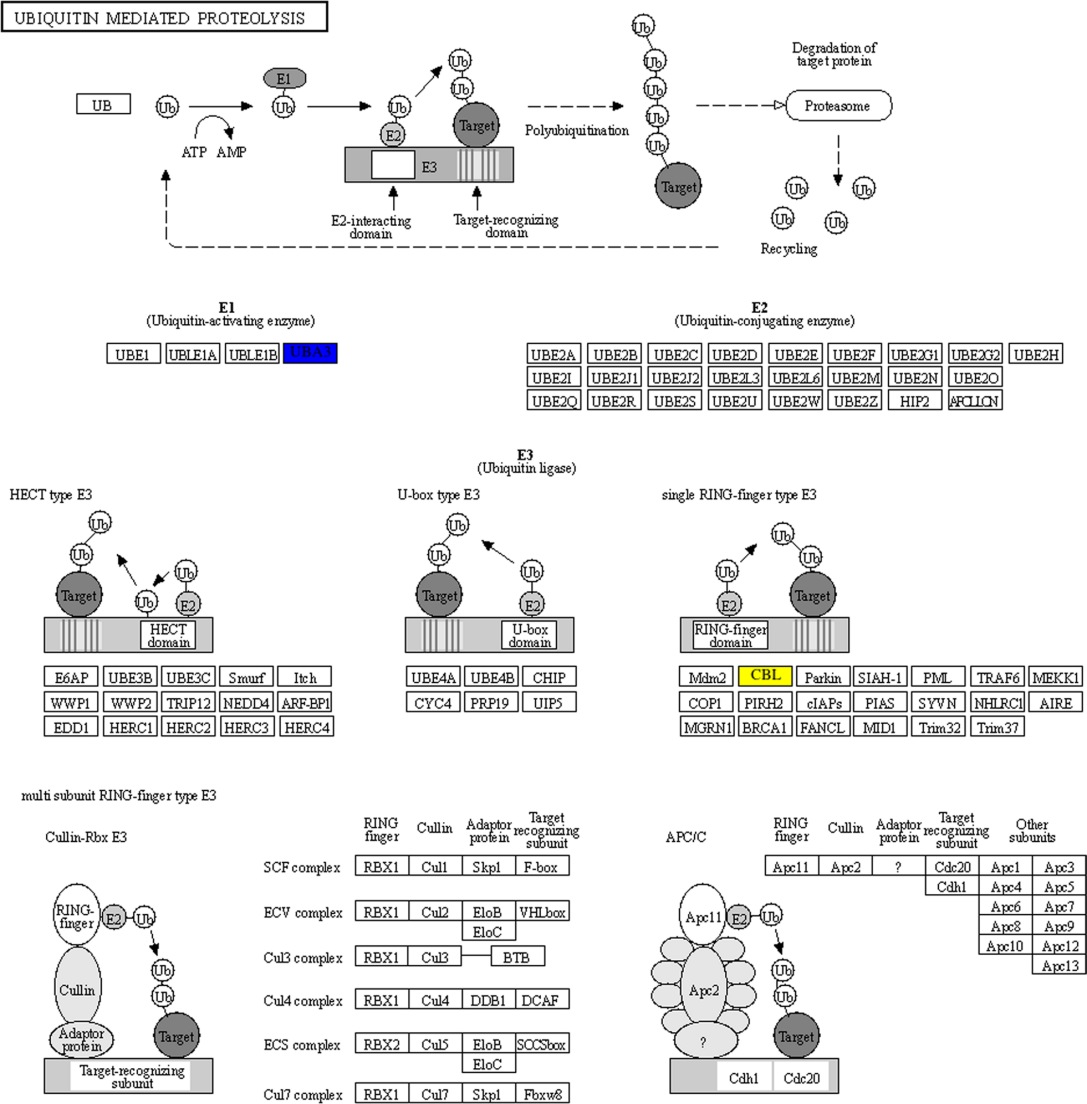
KEGG pathway for ubiquitin-mediated proteolysis. The red square frame indicates upregulated proteins, blue represents downregulated proteins, yellow indicates upregulated genes, and green represents downregulated genes

In the transcriptomic analysis, a total of 7622 differentially expressed genes (DEGs) were identified in adults versus plerocercoids in the transcriptomic analysis, of which 4144 DEGs were upregulated and 3478 were downregulated. The differentially expressed genes in two different developmental stages of *S. mansoni* are listed in Additional file 6: Table S8. To reveal the relationships between differentially expressed genes and proteins, a heatmap was generated by R package correlation analysis. In the correlation between 300 DEGs and 89 DEPs, the heatmap suggested that most DEGs and DEPs showed a positive correlation (*P* < 0.05), such as 40S ribosomal protein S3a was positively correlated with DEGs including tubulin alpha chain, testis-specific, uncharacterized protein, putative aminopeptidase W07G4.4, calmodulin, cAMP-dependent protein kinase type II regulatory subunit, casein kinase I isoform alpha, fructose-bisphosphate aldolase and tubulin alpha chain. Nevertheless, several negative correlations were also observed between the two groups (*P* < 0.05), such as serine/threonine-protein phosphatase showed negative correlation with retinol dehydrogenase 14, ATP binding cassette subfamily B MDR:TAP, uncharacterized protein, carnitine O-palmitoyltransferase 1 and DNA-directed RNA polymerase III subunit RPC8. (Additional file 2: Fig. S3). To further understand those correlated DEGs and DEPs regarding their biological roles, enrichment analysis based on KEGG analysis was performed. Specifically, three overlapping pathways were observed. One pathway was ubiquitin-mediated proteolysis including one upregulated DEG [uncharacterized protein (SERJ2_LOCUS19294)] and one downregulated DEP [NEDD8-activating enzyme E1 catalytic subunit (A0A7M3Q355)] (Fig. 8). The second was phagosomes (Additional file 2: Fig. S4). It was enriched by an upregulated DEG (Tubulin alpha chain) and two downregulated DEGs (V-type proton ATPase proteolipid subunit, tubulin alpha chain and one upregulated DEP). The last one was a spliceosome, which was enriched by one upregulated DEG (Putative lsm1) and one upregulated DEP (RNA helicase) (Additional file 2: Fig. S5). More interestingly, both the combined analysis based on DETs and DEPs and a single analysis using the proteomic data supported that proteins related to the regulation of metabolism and genetic information processing played important roles in the growth and development of *S. mansoni.*

## Discussion

*Spirometra mansoni*, as a neglected medical tapeworm, is among the most hazardous food- and waterborne parasites worldwide [3, 31]. In our previous study, a comparative transcriptomic analysis of the plerocercoid stage and the adult stage of *Spirometra* tapeworm was performed using RNA-seq analysis [11]. Unigenes involved in protein phosphorylation/dephosphorylation and metabolic activities were enriched in plerocercoids and adults, respectively, at the RNA level. However, little is known about global functional protein expression and protein interactions in this tapeworm at the proteomic level. Therefore, in this study, we conducted a comparative proteomic analysis of plerocercoids and adults to continue our last study and to comprehensively reveal the protein expression profiles. In addition, our research provides more valuable clues for understanding the growth and development of this tapeworm.

As an important method, the TMT technique is used for quantitative proteomics and exhibits high throughput and high reproducibility [32, 33]. Here, an integrated approach involving TMT labelling and LC-MS/MS was applied to identify the DEPs in plerocercoids and adults of *S. mansoni*. In total, 3378 proteins were identified, and 3055 proteins were quantified. Compared with other cestodes, the number of identified proteins of the proteome in *S. mansoni* was greater than that in *E. granulosus* (1610 proteins) [19], *H. diminuta* (415 proteins) [16] and *M. corti* (571 proteins) [17]. For *S. mansoni*, the different stages of their life cycle generally involve different environments and living requirements; therefore, some proteins related to certain pathways or enzymes may be unique to these two phases of development and coincide with the requirements and challenges imposed by the different environments [34, 35]. Specifically, 1166 differentially expressed proteins were identified, with 641 up- and 525 downregulated proteins in adults versus plerocercoids, which was similar to our previous RNA-seq study, except that we identified slightly more upregulated genes in adults [11], indicating the accuracy of the quantitative proteomic results. Nevertheless, 860 hypothetical proteins and 306 described proteins were identified among these DEPs, possibly because limited reference omics data were available and few studies have been conducted on this species [36].

To determine the putative functions of DEPs, all identified DEPs were then annotated for GO function and COG classification. According to GO enrichment analysis, among the DEPs in adults versus plerocercoids, the top three enriched BP (translation, glycolytic process and microtubule-based process), MF (ribosome, GTPase activity and ferric iron binding) and CC (ribosome, dynein complex and small ribosomal subunit) categories indicate that most belong to energy metabolism-related and genetic information processing proteins. These annotations cover functions that are important to parasite metabolism, lifestyle and survival [37, 38], and they are also enriched in the annotated proteomes of other parasites, such as *M. corti* and *T. ovis* [23, 39]. The COG pathway enrichment analysis showed that the largest three groups (36% of the DEPs) were associated with "general function prediction only", "translation, ribosomal structure and biogenesis" and "posttranslation modification, protein turnover, chaperones", such as serine/threonine protein phosphatase, arginyl-tRNA synthetase and glutathione transferase. These pathways may be related to parasite growth and development and contribute to their response to stress [40–42]. KEGG pathway analysis is generally used to capture the systematic properties of the inner activities of cells as reference information for the integration and interpretation of high-throughput proteomics datasets [43, 44]. Therefore, we performed KEGG pathway enrichment analysis with the aim of discovering important molecules or pathways that play key roles during *S. mansoni* growth and development. The results suggested that ribosome, glycolysis/gluconeogenesis, lysosome and glutathione metabolism were the top four significantly different pathways, which agrees with the results of GO annotation and COG analysis. The metabolism of glucose is among the significant biochemical processes that provide energy in the form of ATP, which is temporarily stored [45]. In upregulated proteins of adults, the enriched pathways were mainly related to metabolism, such as histidine metabolism and d-glutamine and d-glutamate metabolism, while upregulated proteins of plerocercoids were mainly enriched in catabolism of cellular processes. The results of enriched pathways of upregulated proteins indicated that functional analysis of the corresponding proteins showed stage-specific features and that the metabolism of energy in adults seems to be more activated because of development and sexual reproduction [46, 47].

Through PPI network analysis, we found that six central proteins, including phosphomannomutase, glutathione transferase, malate dehydrogenase, cytoplasmic, 40S ribosomal protein S15, ribosomal protein L15 and 60S acidic ribosomal protein P2c, possessed more than three nodes, indicating that these proteins might be more significant than other proteins [48, 49]. The enzyme phosphomannomutase catalyses the interconversion of mannose-1-phosphate (Man-1-P) and mannose-6-phosphate (Man-6-P) and is an essential step in mannose activation and the biosynthesis of glycoconjugates in all eukaryotes [50]. Glutathione transferases are multifunctional proteins that can protect the cell against exogenously derived compounds (xenobiotics) and toxic endogenously derived compounds by catalysing their conjugation with glutathione (GSH), including secondary products of lipid peroxidation [42]. In addition, several GSH transferases can be used as intracellular transport proteins for a number of ligands [42, 51]. In parasites, cytoplasmic malate dehydrogenase (sMDH) plays major roles in the biosynthesis of highly volatile fatty acids (HVFAs) from carbohydrates through a pathway that involves the production of large quantities of succinate [52]. Ribosomal proteins are components of ribosomes and participate in structural constituents, assembly and protein translation in ribosomes [53]. These key proteins related to metabolism and protein synthesis are significant for the growth and development of parasites and might play key roles in the growth and development of *S. mansoni*.

The composition of the transcriptome and proteome changes throughout the life cycle of most platyhelminthes [54, 55]. Comparing the changes in the composition of the transcriptome and proteome between different life cycle stages of parasites is a promising approach for identifying potentially important differentially expressed genes and proteins as well as exploring complex biological processes and their functions [56, 57]. Therefore, we integrated the transcriptomic and proteomic data of *S. mansoni* to obtain more insightful knowledge about the molecular biology of this neglected tapeworm. Here, we performed KEGG functional enrichment analysis using the combination data of DEGs and DEPs to select proteins that are expressed differentially and find their functions in individuals to achieve a better understanding of bioprocesses. After enriching these differential genes/proteins to KEGG, three common pathways shared by DEPs and DEGs were found, namely, ubiquitin-mediated proteolysis, phagosome and spliceosome, indicating the significance and noteworthy of these pathways in *S. mansoni*. Ubiquitin-mediated proteolysis plays a role in most cellular events, such as cellular contractility, a behaviour of major importance for cell movement and proliferation [58]. Phagosomes are involved in cellular processes, such as the transport and catabolism of lipids and proteins [59, 60]. As a gene regulation mechanism, posttranscriptional regulation has a vital influence on the stage-specific changes in different life stages of parasites. Protein-coding genes need to be transcribed into polycistronic RNA units, which are further processed into individual mRNAs. Spliceosomes play a key role in these processes [61, 62]. Furthermore, five key proteins were detected to be involved in the above three pathways. The first is NEDD8-activating enzyme (NAE) E1, which is involved in ubiquitin-mediated proteolysis. NAE initiates the transfer of the ubiquitin-like protein Nedd8 through an enzymatic cascade to posttranslationally modify target proteins, thus regulating their biological activities in cells [63]. The V-type proton ATPase proteolipid subunit, tubulin alpha chain and tubulin beta chain were detected in the phagosome pathway. The V-type proton ATPase proteolipid subunit participates in many vital processes, such as protein trafficking as a kind of ATPase/ATP synthase [64, 65]. The tubulin alpha chain and tubulin beta chain are components of microtubules, the essential components of the cytoskeleton, and play major roles in several basic cell functions, including phagosome-lysosome fusion. Phagosomes are transported within cells along the cytoskeleton [66]. RNA helicase was detected in the spliceosome pathway. RNA helicases play an essential role in all nucleic acid metabolic processes, and they are ubiquitous enzymes that separate DNA duplexes or unwind secondary structures in ribonucleic acids by utilizing the energy released from ATP hydrolysis. RNA helicases remodel the spliceosome to enable pre-mRNA splicing and play many essential roles in cell development and growth [67, 68]. Although these proteins play important roles in many biological processes, little is known about the characteristics and functions of these proteins in *S. mansoni*. Therefore, further analysis will be necessary to determine the function of these proteins in *S. mansoni*. Additionally, several negative correlations and weak correlations were discovered. This phenomenon may be attributed to multiple factors, including translational efficiency, alternative splicing, mRNA stability, folding, assembly, transport, localization, secretion and degradation [56, 69–71].

## Conclusions

In this study, we integrated transcriptome and proteome analysis of the plerocercoid and adult stages of *S. mansoni* to explore the genetic basis of the growth and development of the neglected medical tapeworm. Our overall proteome analysis provided a rich list of proteins expressed in the plerocercoid and adult stages. In total, 1166 DEPs were screened from two stages, and most DEPs were associated with metabolic activity and genetic information processing. The differences in these proteins between consecutive developmental stages of *S. mansoni* may reflect specific strategies and adaptation mechanisms used by this organism in the process of growth and development. According to the PPI results, six hub proteins are likely involved in regulating the growth and development of *S. mansoni*. Three pathways (ubiquitin-mediated proteolysis, phagosome and spliceosome) and five key proteins (NEDD8-activating enzyme E1 catalytic subunit, V-type proton ATPase proteolipid subunit, tubulin alpha chain, tubulin beta chain, RNA helicase) related to these pathways were identified in the combined analysis of the transcriptome and proteome, indicating significant roles of the pathways and proteins in the growth and development of *S. mansoni*. These molecular and signalling pathways greatly contribute to current genetic resources and further clarify the biological and physiological mechanisms of *Spirometra* tapeworms as well as other related taxa.

### Supplementary Information

**Additional file 1.** The comparative analysis of contaminants about the *Spirometra mansoni* in the cRAP database.

**Additional file 2: Table S2.** Primers sequences designed for RT-qPCR. **Table S5.** Top 30 DEPs with description associated with the prouduction, transport and metabolism of nutrients in *Spirometra mansoni*. **Table S6.** Protein interaction network of proteins in metabolic pathways. **Table S7.** Protein interaction network of proteins in genetic information processing. **Figure S1.** Protein quantification results. Heatmaps of all the identified proteins. Red indicates significant upregulation of protein expression levels, significant downregulation in blue, and white represents no statistically significant protein expression levels. **Figure S2.** **a** The top 20 GO enrichments of significantly upregulated proteins in adults. **b** The top 20 GO enrichments of significantly upregulated genes in plerocercoids. **Figure S3.** Integrated analysis of transcriptome and proteome in plerocercoid and adult. Comparison of differences in transcript and protein expression levels. **Figure S4.** KEGG pathway for phagosome. The red square frame indicates upregulated proteins, blue represents downregulated proteins, yellow indicates upregulated genes and green represents downregulated genes. **Figure S5.** KEGG pathway for spliceosome. The red square frame indicates upregulated proteins, blue represents downregulated proteins, yellow indicates upregulated genes and green represents downregulated genes.

**Additional file 3: Table S1.** The full data of UP000550142.

**Additional file 4: Table S3.** All identified proteins in two different developmental stages of *Spirometra mansoni*.

**Additional file 5: Table S4.** The differentially expressed proteins in two different developmental stages of *Spirometra mansoni*.

**Additional file 6: Table S8.** The differentially expressed genes in two different developmental stages of *Spirometra* *mansoni*.

## Data Availability

The data supporting the conclusions of this article are included within the article.

## Abbreviations

DEP: Differentially expressed protein
FDR: False discovery rate
Uniprot: Universal protein database
TMT: Tandem mass tag
LC–MS/MS: Liquid chromatography coupled with tandem mass spectrometry
GO: Gene Ontology
KEGG: Kyoto Encyclopedia of Genes and Genomes
COG/KOG: Clusters of Orthologous Groups/Clusters of euKaryotic Orthologous Groups
BP: Biological process
CC: Cellular component
MF: Molecular function
PPI: Protein-protein interaction
qRT-PCR: Quantitative real-time PCR
GAPDH: Glyceraldehyde-3-phosphate dehyderogenase
