# Supplementary material for: Integrated transcriptomic and proteomic analyses of plerocercoid and adult Spirometra mansoni reveal potential important pathways in the development of the medical tapeworm
Source: Parasit Vectors. 2023 Sep 5;16:316. doi: 10.1186/s13071-023-05941-8 (PMC10481575; doi:10.1186/s13071-023-05941-8)
Supplement: Supplementary file 2 — Additional file 2: Table S2. Primers sequences designed for RT-qPCR. Table S5. Top 30 DEPs with description associated with the prouduction, transport and metabolism of nutrients in Spirometra mansoni. Table S6. Protein interaction network of proteins in metabolic pathways. Table S7. Protein interaction network of proteins in genetic information processing. Figure S1. Protein quantification results. Heatmaps of all the identified proteins. Red indicates significant upregulation of protein expression levels, significant downregulation in blue, and white represents no statistically significant protein expression levels. Figure S2. a The top 20 GO enrichments of significantly upregulated proteins in adults. b The top 20 GO enrichments of significantly upregulated genes in plerocercoids. Figure S3. Integrated analysis of transcriptome and proteome in plerocercoid and adult. Comparison of differences in transcript and protein expression levels. Figure S4. KEGG pathway for phagosome. The red square frame indicates upregulated proteins, blue represents downregulated proteins, yellow indicates upregulated genes and green represents downregulated genes. Figure S5. KEGG pathway for spliceosome. The red square frame indicates upregulated proteins, blue represents downregulated proteins, yellow indicates upregulated genes and green represents downregulated genes. [file 13071_2023_5941_MOESM2_ESM.docx]

**Supplementary materials**

**Integrated transcriptomic and proteomic analyses of plerocercoid and adult *Spirometra mansoni* reveal three potential important pathways in the development of the medical tapeworm**

**Table S2.** Primers sequences designed for RT-qPCR.

**Table S5.** Top 30 DEPs with description associated with the prouduction, transport and metabolism of nutrients in *S. mansoni.*

**Table S6.** Protein interaction network of proteins in metabolic pathways.

**Table S7.** Protein interaction network of proteins in genetic information processing.

**Figure S1.** Protein quantification results. Heatmaps of all the identified proteins. Red indicates significant upregulation of protein expression levels, significant downregulation in blue, and white represents no statistically significant protein expression levels.

**Figure S2.** (a) The top 20 GO enrichments of significantly upregulated proteins in adults. (b) The top 20 GO enrichments of significantly upregulated genes in plerocercoids.

**Figure S3.** Integrated analysis of transcriptome and proteome in plerocercoid and adult. Comparison of differences in transcript and protein expression levels.

**Figure S4.** KEGG pathway for phagosome. The red square frame indicates upregulated proteins, blue represents downregulated proteins, yellow indicates upregulated genes and green represents downregulated genes.

**Figure S5.** KEGG pathway for spliceosome. The red square frame indicates upregulated proteins, blue represents downregulated proteins, yellow indicates upregulated genes and green represents downregulated genes.

**Table S2.** Primers sequences designed for RT-qPCR.

| **Stage** | **Protein description** | **Gene name** | **Primer Sequence (5' to 3')** | **Product size (bp)** |
| --- | --- | --- | --- | --- |
| Adult | L-lactate dehydrogenase | SERJ2_LOCUS16733 | F-CGGTGCTCCTGGTTGTAT  R-ATCACCGTGTTCGCCTAT | 194 |
|  | Fructose-bisphosphate aldolase | SERJ2_LOCUS11901 | F-GGCACATTGCTCAAACCG  R-TTTGGATTGTTGCGAGGG | 91 |
|  | Reticulon-like protein | SERJ2_LOCUS22103 | 1. CTGGCGATGTCGTTGTTC   R-GATGGCCTCGTCGATTTG | 266 |
|  | Proline dehydrogenase | SERJ2_LOCUS28270 | F-GTGCTCTTTGGGCTGGTT  R-AGCTTCGGACTCGGGAAT | 189 |
|  | Galactosylgalactosylxylosylprotein 3-beta-glucuronosyltransferase | SERJ2_LOCUS21092 | F-TCAGACCCAGGCGGAAGT  R-GCTCATTGGCATGGACGT | 108 |
|  | Translocation protein SEC62 | SERJ2_LOCUS13689 | F-AGAAGTCGGAGGCTAAATC  R-GAACACTTGAGCTTCCGTAT | 113 |
| plerocercoid | Glutathione transferase | SERJ2_LOCUS4954 | F-GCCATCAGGAAATACAATC  R-CTTCTCCGTTATGTAGGC | 217 |
|  | Triosephosphate isomerase | SERJ2_LOCUS16521 | F-CCGACCTTGACCCTAACG  R-CTGGCAACCGCAATCCTT | 176 |
|  | Vacuolar protein sorting-associated protein 28 homolog | SERJ2_LOCUS14814 | F-TGTTGGCAAGTGTATCGC  R-GTGGCAGAAGGTTAAGTCG | 143 |
|  | Phosphomannomutase | SERJ2_LOCUS18729 | 1. TGCTTTGCCTATTTGACG   R-TGCTTAGATCGGACCCAC | 132 |
|  | Calmodulin | SERJ2_LOCUS10404 | F-TCGACTTCCCTGAGTTCC  R-CGTATCATCTCGTCCACCT | 190 |
|  | Ferritin | SERJ2_LOCUS25042 | F-GGAAACAGCCCTCAAGAT  R-GACGTACCCGGAAATCTG | 157 |
| Reference gene | glyceraldehyde-phosphatedehydrogenase (GADPH) |  | F-AGCAACCTCGTTGATGTCGT  R-TGAATTGACCGTGGGTGGAG | 97 |

**Table S5.** DEPs with description associated with the prouduction, transport and metabolism of nutrients of *S. mansoni.*

| **Accession** | **Description** | **FC (CC_YC)** | **pValue (CC_YC)** | **Sig (CC_YC)** |
| --- | --- | --- | --- | --- |
| A0A7M3Q3I4 | Adenylosuccinate synthetase | 0.292868 | 0.000001 | -1 |
| A0A7M3PZQ3 | Isocitrate dehydrogenase [NAD] subunit, mitochondrial | 0.302934 | 0.000001 | -1 |
| A0A7M3PWE7 | ADP-ribosyl cyclase/cyclic ADP-ribose hydrolase | 0.330307 | 0 | -1 |
| A0A7M3R4D4 | V-type proton ATPase subunit | 0.332865 | 0.00004 | -1 |
| A0A7M3RHZ3 | Oxoglutarate dehydrogenase (succinyl-transferring) | 2.622993 | 0.000014 | 1 |
| A0A7M3RTM9 | Beta-galactosidase | 0.400202 | 0.000031 | -1 |
| A0A7M3R222 | Triosephosphate isomerase | 0.409779 | 0 | -1 |
| A0A7M3R3A0 | L-lactate dehydrogenase | 2.380184 | 0.000073 | 1 |
| A0A7M3PYD2 | Dihydrolipoyllysine-residue succinyltransferase component of 2-oxoglutarate dehydrogenase complex, mitochondrial | 2.336336 | 0.000001 | 1 |
| A0A7M3RJK1 | Triosephosphate isomerase | 0.431613 | 0.000002 | -1 |
| A0A7M3RUJ8 | Glutamate dehydrogenase (NAD(P)(+)) | 2.296487 | 0.00014 | 1 |
| A0A7M3RZR3 | Proline dehydrogenase | 2.278584 | 0.000115 | 1 |
| A0A7M3RFA3 | Phosphomannomutase | 0.452574 | 0.000013 | -1 |
| A0A7M3RFG4 | Heparan-sulfate 6-O-sulfotransferase | 2.197695 | 0.000073 | 1 |
| A0A7M3R0S1 | 2-phospho-D-glycerate hydro-lyase | 2.129119 | 0.000003 | 1 |
| A0A7M3QAD3 | FACT complex subunit | 2.113195 | 0.000026 | 1 |
| A0A7M3QUD2 | Arginase | 2.092182 | 0.000321 | 1 |
| A0A7M3R6W9 | Malate dehydrogenase | 2.086693 | 0.000001 | 1 |
| A0A7M3RDB8 | Acetyltransferase component of pyruvate dehydrogenase complex | 2.068084 | 0.000002 | 1 |
| A0A7M3QN57 | Glyceraldehyde-3-phosphate dehydrogenase | 2.048615 | 0.000001 | 1 |
| A0A7M3RUS2 | L-lactate dehydrogenase | 0.498647 | 0.000014 | -1 |
| A0A7M3RE77 | Propanoyl-CoA:carbon dioxide ligase subunit alpha | 1.979046 | 0.000001 | 1 |
| A0A7M3PY64 | Beta-hexosaminidase | 0.506131 | 0.000002 | -1 |
| A0A7M3VJT9 | Triosephosphate isomerase | 0.513191 | 0.000002 | -1 |
| A0A7M3R503 | Phosphohydroxythreonine aminotransferase | 0.5147 | 0.000644 | -1 |
| A0A7M3QRP4 | Glyceraldehyde-3-phosphate dehydrogenase | 0.520792 | 0.000001 | -1 |
| A0A7M3R8X6 | Oxoglutarate dehydrogenase (succinyl-transferring) | 1.918284 | 0.000002 | 1 |
| A0A7M3RFX8 | Pyrroline-5-carboxylate reductase | 1.913066 | 0.000001 | 1 |
| A0A7M3Q176 | ATP synthase subunit O, mitochondrial | 1.907674 | 0.000012 | 1 |
| A0A7M3Q8T8 | Pyruvate carboxylase | 1.905347 | 0.000002 | 1 |

**Table S6.** Protein interaction network of proteins in metabolic pathways.

| Pro1 | Description | Pro2 | Description | Diff1 | Diff2 | Combined_score |
| --- | --- | --- | --- | --- | --- | --- |
| A0A7M3RPH5_SPIER | Glutathione transferase | A0A7M3Q4K1_SPIER | Glutathione transferase | -1 | -1 | 0.989 |
| A0A7M3QQR0_SPIER | Glutathione transferase | A0A7M3Q4K1_SPIER | Glutathione transferase | -1 | -1 | 0.989 |
| A0A7M3RFA3_SPIER | Phosphomannomutase | A0A7M3QVA0_SPIER | Phosphotransferase | -1 | -1 | 0.921 |
| A0A7M3RHK2_SPIER | V-type proton ATPase proteolipid subunit | A0A7M3RNM5_SPIER | ATP synthase subunit alpha | -1 | 1 | 0.919 |
| A0A7M3Q0W2_SPIER | L-lactate dehydrogenase | A0A7M3QN57_SPIER | Glyceraldehyde-3-phosphate dehydrogenase | -1 | 1 | 0.701 |
| A0A7M3PY64_SPIER | Beta-hexosaminidase | A0A7M3QF48_SPIER | GlcNAc kinase | -1 | 1 | 0.927 |
| A0A7M3RUS2_SPIER | L-lactate dehydrogenase | A0A7M3QN57_SPIER | Glyceraldehyde-3-phosphate dehydrogenase | -1 | 1 | 0.701 |
| A0A7M3RXL5_SPIER | Glutathione transferase | A0A7M3Q4K1_SPIER | Glutathione transferase | -1 | -1 | 0.989 |
| A0A7M3QAB2_SPIER | Glucose-6-phosphate isomerase | A0A7M3RUS2_SPIER | L-lactate dehydrogenase | -1 | -1 | 0.937 |
| A0A7M3QUD2_SPIER | Arginase | A0A7M3RNA2_SPIER | Glutamate dehydrogenase | 1 | 1 | 0.717 |
| A0A7M3QVN6_SPIER | Fructose-bisphosphatase | A0A7M3RFA3_SPIER | Phosphomannomutase | 1 | -1 | 0.805 |
| A0A7M3RB85_SPIER | Acyl carrier protein |  |  |  |  |  |
| A0A7M3QRS4_SPIER | Fructose-bisphosphate aldolase | A0A7M3Q3N0_SPIER | Malate dehydrogenase, cytoplasmic | 1 | 1 | 0.71 |
| A0A7M3Q3N0_SPIER | Malate dehydrogenase, cytoplasmic |  |  |  |  |  |
| A0A7M3RNA2_SPIER | Glutamate dehydrogenase | A0A7M3RFA3_SPIER | Phosphomannomutase | 1 | -1 | 0.703 |
| A0A7M3QRP6_SPIER | Fructose-bisphosphate aldolase | A0A7M3Q3N0_SPIER | Malate dehydrogenase, cytoplasmic | 1 | 1 | 0.71 |
| A0A7M3RNM5_SPIER | ATP synthase subunit alpha |  |  |  |  |  |
| A0A7M3RWF4_SPIER | Fructose-bisphosphate aldolase | A0A7M3Q3N0_SPIER | Malate dehydrogenase, cytoplasmic | 1 | 1 | 0.71 |
| A0A7M3Q813_SPIER | Glutamate dehydrogenase | A0A7M3RFA3_SPIER | Phosphomannomutase | 1 | -1 | 0.703 |
| A0A7M3Q176_SPIER | ATP synthase subunit O, mitochondrial | A0A7M3RB85_SPIER | Acyl carrier protein | 1 | 1 | 0.937 |
| A0A7M3QN57_SPIER | Glyceraldehyde-3-phosphate dehydrogenase |  |  |  |  |  |
| A0A7M3RUJ8_SPIER | Glutamate dehydrogenase (NAD(P)(+)) | A0A7M3RFA3_SPIER | Phosphomannomutase | 1 | -1 | 0.703 |

**Table S7.** Protein interaction network of proteins in genetic information processing.

| Pro1 | Description | Pro2 | Description | Diff1 | Diff2 | Combined_score |
| --- | --- | --- | --- | --- | --- | --- |
| A0A7M3Q9T2_SPIER | Ribosomal protein L15 | A0A7M3QU30_SPIER | 40S ribosomal protein S15 | 1 | 1 | 0.999 |
| A0A7M3Q9L8_SPIER | 40S ribosomal protein S4 |  |  |  |  |  |
| A0A7M3QU61_SPIER | Translocation protein SEC62 | A0A7M3RB26_SPIER | Signal recognition particle 54 kDa protein | 1 | 1 | 0.85 |
| A0A7M3Q9T7_SPIER | 60S ribosomal protein L35a | A0A7M3RBW7_SPIER | 60S acidic ribosomal protein P2 | 1 | 1 | 0.998 |
| A0A7M3QJ75_SPIER | 60S ribosomal protein L17 | A0A7M3Q066_SPIER | 60S ribosomal protein L21 | 1 | 1 | 0.998 |
| A0A7M3RBA1_SPIER | 40S ribosomal protein S2 |  |  |  |  |  |
| I6T8V1_SPIER | 60S ribosomal protein L27e | A0A7M3QRE8_SPIER | Ribosomal protein | 1 | 1 | 0.999 |
| A0A7M3RB26_SPIER | Signal recognition particle 54 kDa protein |  |  |  |  |  |
| A0A7M3Q950_SPIER | 60S ribosomal protein L29 | A0A7M3RBW7_SPIER | 60S acidic ribosomal protein P2 | 1 | 1 | 0.997 |
| A0A7M3R840_SPIER | 60S ribosomal protein L12 | A0A7M3Q9T2_SPIER | 60S ribosomal protein L35a | 1 | 1 | 0.998 |
| A0A7M3RBW7_SPIER | 60S acidic ribosomal protein P2 | A0A7M3RBA1_SPIER | 40S ribosomal protein S2 | 1 | 1 | 0.994 |
| A0A7M3RC38_SPIER | 40S ribosomal protein S23 | A0A7M3Q1Y5_SPIER | 60S ribosomal protein L9 | 1 | 1 | 0.987 |
| A0A7M3R5G1_SPIER | 40S ribosomal protein S6 | A0A7M3Q9T2_SPIER | Ribosomal protein L15 | 1 | 1 | 0.999 |
| A0A7M3QRE8_SPIER | Ribosomal protein |  |  |  |  |  |
| A0A7M3Q066_SPIER | 60S ribosomal protein L21 | A0A7M3Q8W9_SPIER | 60S ribosomal protein L28 | 1 | 1 | 0.999 |
| A0A7M3QDQ4_SPIER | 40S ribosomal protein S18 | A0A7M3RB26_SPIER | Signal recognition particle 54 kDa protein | 1 | 1 | 0.952 |
| A0A7M3Q8W9_SPIER | 60S ribosomal protein L28 | A0A7M3Q9L8_SPIER | 40S ribosomal protein S4 | 1 | 1 | 0.998 |
| A0A7M3Q1Y5_SPIER | 60S ribosomal protein L9 |  |  |  |  |  |
| A0A7M3R087_SPIER | 40S ribosomal protein S20 | A0A7M3QU30_SPIER | 40S ribosomal protein S15 | 1 | 1 | 0.999 |
| A0A7M3QU30_SPIER | 40S ribosomal protein S15 | A0A7M3QRE8_SPIER | Ribosomal protein | 1 | 1 | 0.998 |
| A0A7M3PUT0_SPIER | 60S ribosomal protein L8 | A0A7M3Q9T2_SPIER | Ribosomal protein L15 | 1 | 1 | 0.999 |
| A0A7M3RPJ5_SPIER | 40S ribosomal protein S9 | A0A7M3QU30_SPIER | 40S ribosomal protein S15 | 1 | 1 | 0.999 |


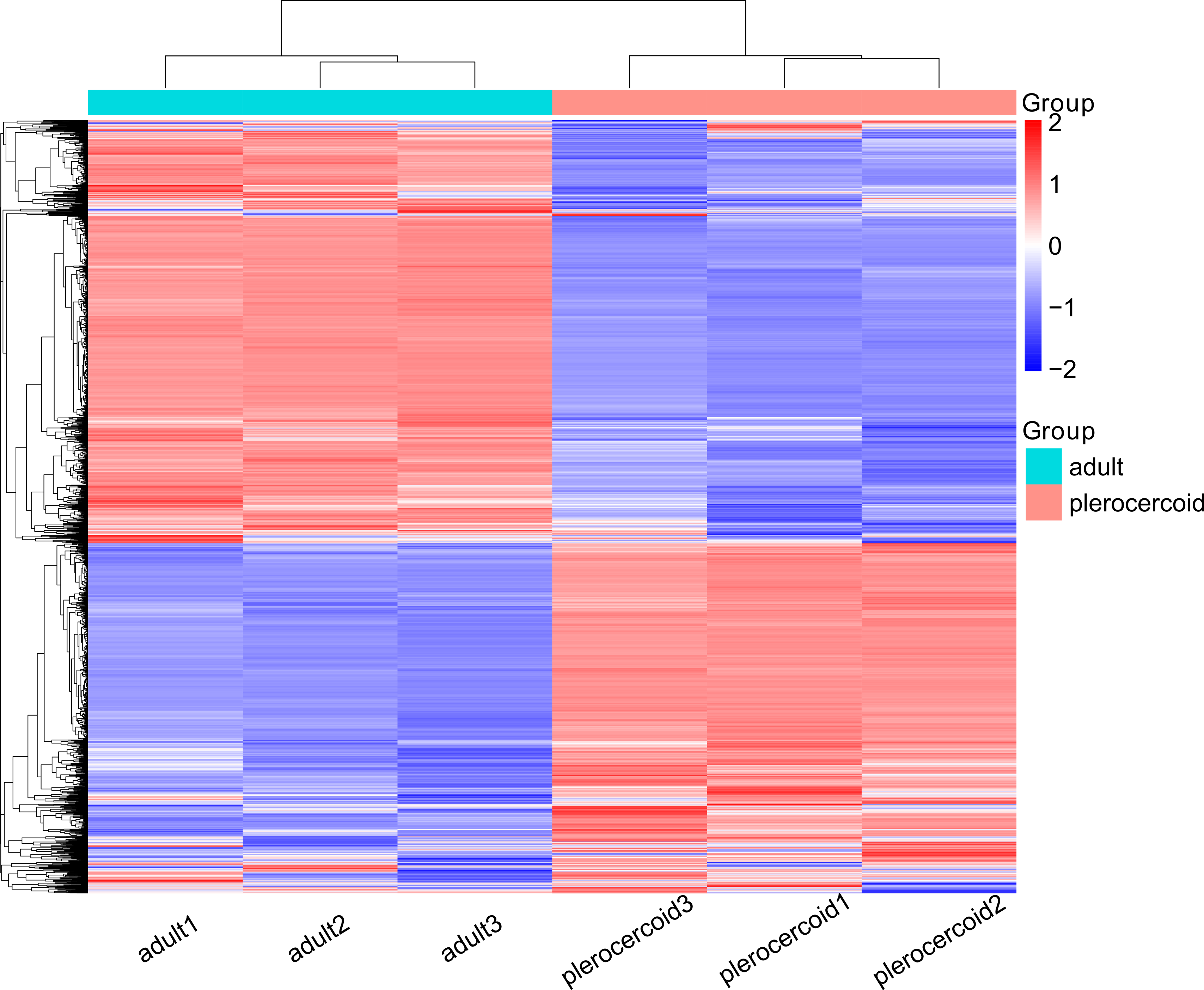


**Figure S1.** Protein quantification results. Heatmaps of all the identified proteins. Red indicates significant upregulation of protein expression levels, significant downregulation in blue, and white represents no statistically significant protein expression levels.


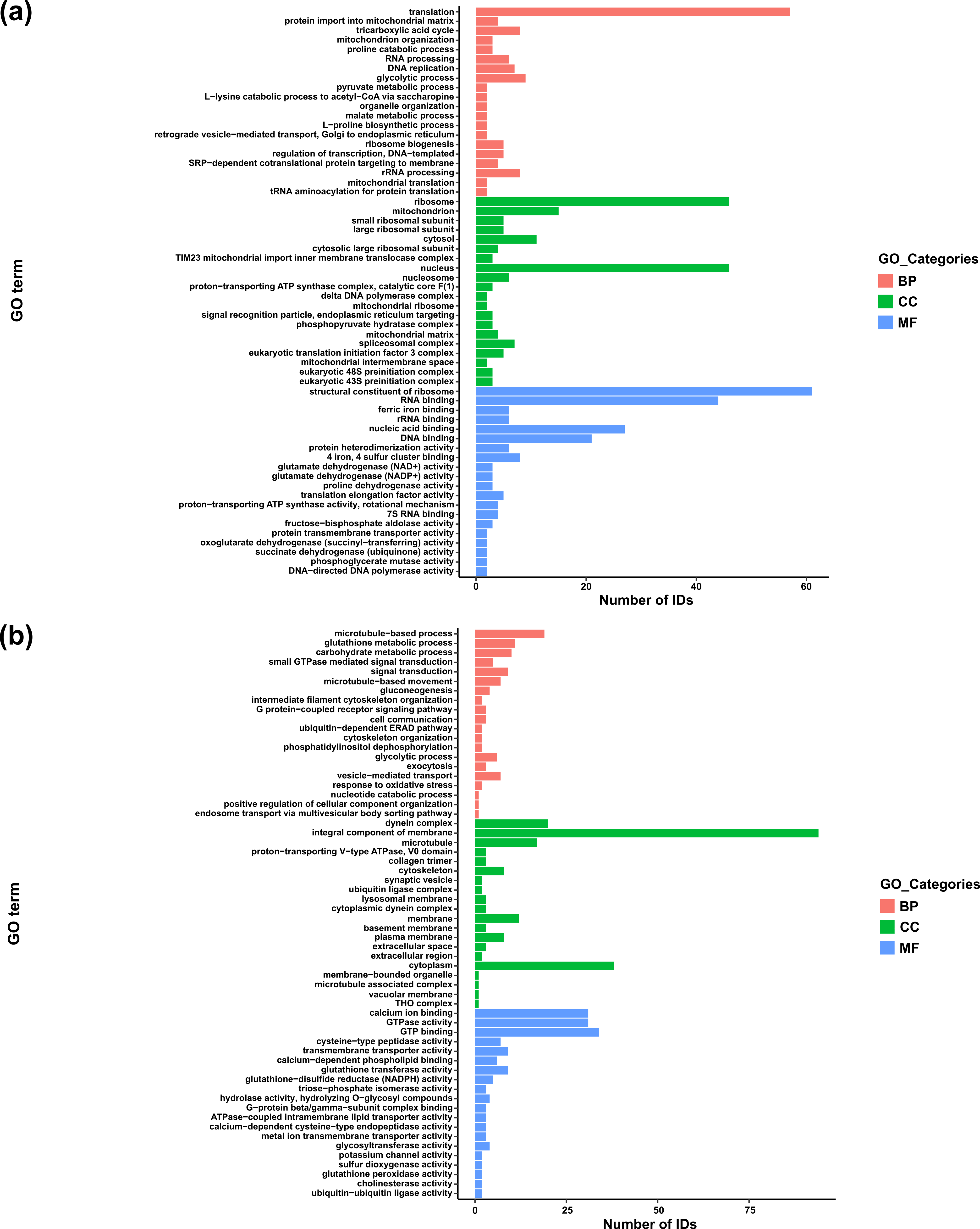


**Figure S2.** GO functional enrichment for the differentially expressed proteins. (a) The top 20 GO enrichments of significantly upregulated proteins in adults. (b) The top 20 GO enrichments of significantly upregulated genes in plerocercoids.


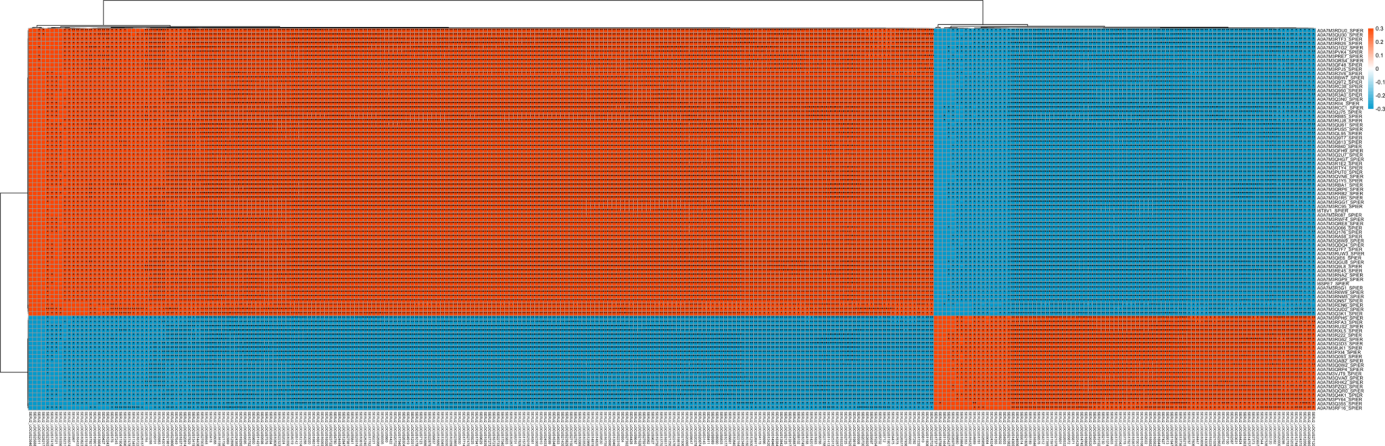


**Figure S3.** Integrated analysis of transcriptome and proteome in plerocercoid and adult. Comparison of differences in transcript and protein expression levels. Red and blue indicate positive and negative correlations, respectively. *P* < 0.05 represents significance, *P* < 0.01 represents high significance, and *P* < 0.001 represents extreme significance. The X-axis indicates DEGs. The Y-axis indicates DEPs.


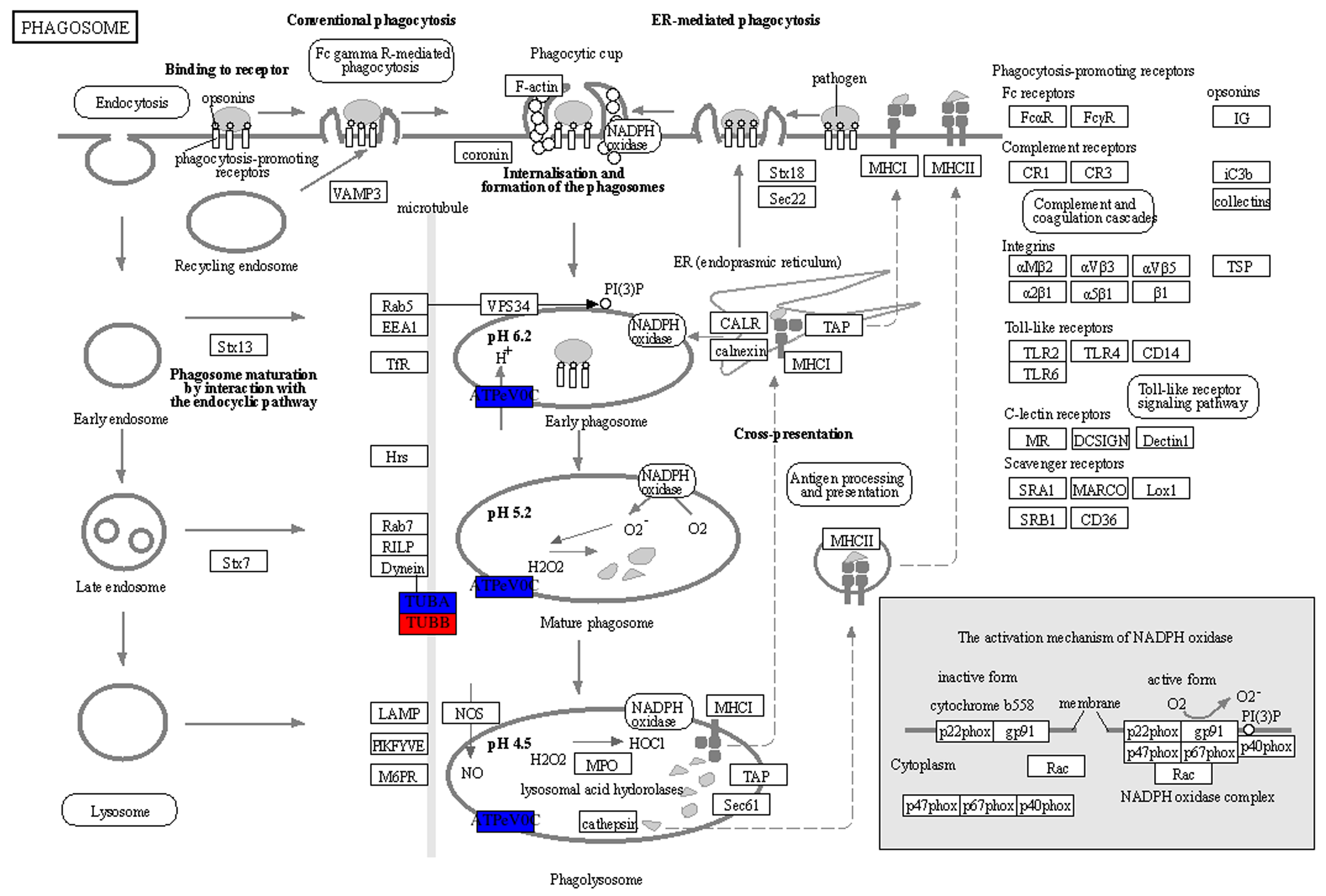


**Figure S4.** KEGG pathway for phagosome. The red square frame indicates upregulated proteins, blue represents downregulated proteins, yellow indicates upregulated genes and green represents downregulated genes.


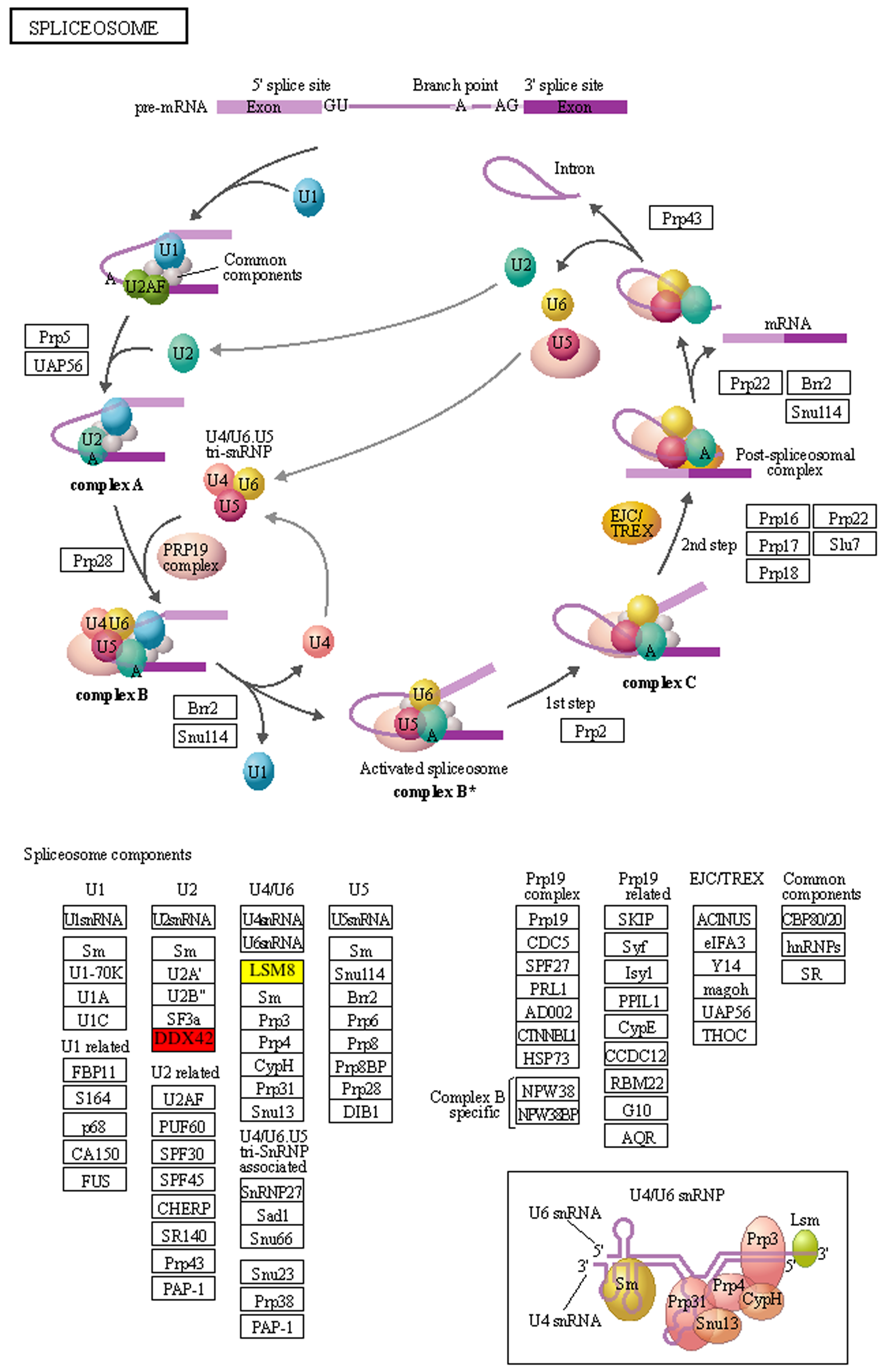


**Figure S5.** KEGG pathway for spliceosome. The red square frame indicates upregulated proteins, blue represents downregulated proteins, yellow indicates upregulated genes and green represents downregulated genes.
